# Comparison of Personal, Social and Academic Variables Related to University Drop-out and Persistence

**DOI:** 10.3389/fpsyg.2016.01610

**Published:** 2016-10-18

**Authors:** Ana Bernardo, María Esteban, Estrella Fernández, Antonio Cervero, Ellián Tuero, Paula Solano

**Affiliations:** ^1^Psychology Department, University of OviedoOviedo, Spain; ^2^Department of Education, University of OviedoOviedo, Spain

**Keywords:** higher education, university drop-out, academic performance, academic adaptation, social adaptation

## Abstract

Dropping out of university has serious consequences not only for the student who drops out but also for the institution and society as a whole. Although this phenomenon has been widely studied, there is a need for broader knowledge of the context in which it occurs. Yet research on the subject often focuses on variables that, although they affect drop-out rates, lie beyond a university’s control. This makes it hard to come up with effective preventive measures. That is why a northern Spanish university has undertaken a ex post facto holistic research study on 1,311 freshmen (2008/9, 2009/10, and 2010/11 cohorts). The study falls within the framework of the ALFA-GUIA European Project and focuses on those drop-out factors where there is scope for taking remedial measures. This research explored the possible relationship of degree drop-out and different categories of variables: variables related to the educational stage prior to university entry (path to entry university and main reason for degree choice), variables related to integration and coexistence at university (social integration, academic integration, relationships with teachers/peers and value of the living environment) financial status and performance during university studies (in terms of compliance with the program, time devoted to study, use of study techniques and class attendance). Descriptive, correlational and variance analyses were conducted to discover which of these variables really distinguish those students who drop-out from their peers who complete their studies. Results highlight the influence of vocation as main reason for degree choice, path to university entry, financial independency, social and academic adaptation, time devoted to study, use of study techniques and program compliance in the studied phenomenon.

## Introduction

Dropping out of higher education is a global phenomenon and it affects virtually all universities (UNESCO, 2004). That is why higher education institutions have researched the kinds of drop-outs, their causes and consequences ever since the early 20th century, and in particular since the 1970s. Durán-Aponte and Pujol (2012) argue that university drop-outs can be classified under one of three heads: voluntary (voluntary or forced drop-out); temporary (whether initial, early or late); scope (internal, institutional or from the education system). However, research currently under way on the phenomenon tends to focus on initial or early voluntary drop-out (that is to say, during the first year of university). That is because this is when most drop-outs tend to occur (Castaño et al., 2004; Willcoxson, 2010; Belloc et al., 2011). Also, for practical reasons most studies focus on internal drop-outs (or change of degree) and institutional drop-outs (where students leave the university concerned but do not necessarily stop studying, whether at a university or other institution). Practical reasons lie behind this focus, especially with regard to sample identification. Such studies cover a wide range of variables (a holistic approach) in order to avoid the biases that were once common.

Detailed study of the factors involved in university drop-out has both given rise to different explanatory models of the phenomenon and revealed its complexity. Some models focus solely on the possible influence of economic variables (Jensen, 1981; Donoso and Schiefelbein, 2007). Other models focus on the various psychological characteristics of students who drop-out (Fishbein and Ajzen, 1977; Belloc et al., 2011). Yet others stress the influence of sociological factors that go beyond the individual (Pincus, 1980), or that affect the education institution itself (Kamers, 1971) or on the interaction between these two (Tinto, 1975). All models look at variables that may explain drop-out and shed light on the phenomenon. That said, at present one of the most commonly applied ones is a reformulation of Tinto’s (1993) adaptive explicative model. This model highlights the importance of characteristics pre-dating university entry and variables such as background, and student adaptation to the institution’s social and academic atmosphere as factors determining student drop-out. This model has been criticized for failing to take into account the cultural diversity of students (Guiffrida, 2006) or variables outside the academic context such as family involvement (Bean, 1983). Notwithstanding these criticisms, the variables included by Tinto in his model seem to carry weight in studies regardless of the context in which they were carried out. This is especially true in those covering the first year of university (Tinto, 2001; Upcraft et al., 2004). On the other hand, learning theorists believe that a student’s commitment to his studies and ability to tackle tasks in a strategic fashion are important variables in academic performance (Azevedo et al., 2010; Broadbent and Poon, 2015). They argue that these variables bear heavily on students deciding whether to stay on at university or to drop-out (Arco-Tirado et al., 2011). Students’ academic adaptation to the university setting thus assumes great importance in the decision to either continue one’s studies or to drop-out. It seems reasonable to think (and has been shown to be true) that it is the students who fail at university that drop-out, not those that succeed (Araque et al., 2009).

Of the drop-out related variables falling under the head of *background*, it seems that the student’s academic track record (e.g., matriculation grade; Smith and Naylor, 2001; Belloc et al., 2011; Burillo et al., 2011) and the student’s financial possibilities are constantly found to be relevant factors. Some research shows that pre-university training can play a role in fostering continuation and completion of the student’s academic studies. Thus some university entrance options (Corominas, 2001; Rodrigo et al., 2012) are associated with higher drop-out rates than others. Reserved place schemes (e.g., vocational training or for those over the age of 25/45) stand out in this respect. This seems to be because students entering through reserved place schemes have different *backgrounds* from those entering straight from school. Here, students joining from school are more likely to complete their studies than those that do not (Lassibille and Navarro, 2009). Similarly, student performance at this stage lays the foundations for future academic attainment. This is because academic grades prior to entry are a good predictor of university performance — something corroborated by Casaravilla et al. (2012) — and thus of the likelihood of a student dropping out. However, one must also take into account the link between choice of degree and reasons for dropping out. As Duncan (2006) noted, this is because informed choice of degree is a predictor of both switching studies and of drop-out from higher education (a variable that combines both matriculation grade and motivational aspects). Here, we should bear in mind that although students may know and wish to entry in a particular degree, a limited availability of places and the requirements of the institution (ej. outstanding academic performance during high school) often prevent them from getting enrolled in their first choice. Not surprisingly, students who have to make do with another choice of degree are more likely to drop-out. In fact, 80% of students who drop-out of certain degree programs had not taken them as their first choice. This was so because either the student’s matriculation grade was too low to get their first choice or factors other than student motivation played a role (Cabrera et al., 2006b; Elias, 2008; Burillo et al., 2011).

In addition to the student’s academic background, financial support is also a constant factor. Students’ financial circumstances and the opportunity cost of undertaking university studies (Chen, 2008) play a role. Students who depend on their own slender resources at university and especially those doing a full-time job during their studies are the ones who are likeliest to drop-out (Elias, 2008; Goldenhersh et al., 2011; Esteban et al., 2016).

While these variables have been shown to be highly relevant, they only partly predict university drop-outs. That is because (as with academic achievement), dropping out is a complex phenomenon. Accordingly, other variables need to be taken into account to explain why students facing similar risks and challenges (financial ones, for instance) and taking the same degrees still manage to graduate (Landry, 2003). Given this, the student’s social adaptation to university, his motivation, commitment and ability to meet academic demands, could be the answer. There are many variables that influence a student’s decision on whether to drop-out or to continue studying — a point noted by Tinto (2006). Some of these variables lie beyond the university’s control. An example here might be the cultural level of the student’s family. So while we concede the theoretical interest of analyzing all aspects bearing on dropping out of university, in this paper we shall focus on those where universities have a chance of making a difference.

University study habits and techniques are linked to both academic performance and student drop-out (Antoni, 2003; Cabrera et al., 2006a). Given the results obtained by Vermetten et al. (1999) and Schmeck (2013), we acknowledge that the most suitable study techniques may vary with the kind of academic training imparted and the student’s preferred learning styles. Depending on the degree chosen, the student’s study techniques may or may not be those required for successful completion of the course. Hence the need to detect mismatches between student’s study techniques and academic requirements, and to take remedial action are necessary (Hernández et al., 2015). In this regard, regular class attendance makes it easier to freshmen to adapt and develop their skills, in order to match the requirements of their particularstudy program, promoting a good academic progress at university (Rodríguez and Herrera, 2009). Regular class attendance also facilitates social contacts, helping to forge links among students, parents, faculty, and other university staff. Such relationships not only foster students’ social and academic adaptation but also help keep students in the degree program (Tinto, 1997). Student support services play a particularly important role in this regard. However, the results may be mediated by the teaching methodology and teaching method, as Braxton et al. (2000) have highlighted. These variables are highly relevant. It therefore behooves universities to delve into them so that they can improve their teaching and organizational methods. Here, universities cannot shirk their responsibilities by laying all the blame for drop-outs on student fecklessness — a point made by Tinto (2006). Instead, universities should strive harder to meet students’ needs. One should not blithely assume that a student who drops out does so because he is poorly motivated, does not work hard enough or lacks ability. Nor should such arguments be taken as an excuse for the university to wash its hands of the situation. Instead, the university must grasp the risk factors so that the right remedial measures can be taken. The university should work with both the student and others, providing as many tools as possible to ensure students graduate.

One should also bear in mind the longitudinal dimension of dropping out — a point stressed by Tinto (1988). In order to explain different kinds of drop-outs, regarding on the moment when the student makes this decision. Tinto (1988) draws on anthropological studies on trive rites of passage, arguing that access to higher education is comparable to these ancient rites, symbolizing the transition of individuals from one social group to another (a process described by Van Gennep, 1960). Here, it is necessary to to recognize feelings of isolation and weakness, similar to those described by Durkheim (1954) under the term “anomie.”

Given the plethora of research studies undertaken to date and for diverse purposes (descriptive, explanatory, predictive, for improvement), one wonders whether further contributions to knowledge are needed in this field. Nevertheless, studying which factors affect dropping out in every cultural context is vital if one is to come up with effective, well-targeted counter-measures. That is because students’ circumstances and educational levels vary among countries and the regions within them (Willcoxson et al., 2011). As Lamb et al. (2010) state, some educational systems are more effective than others at hanging on to students and making sure they graduate.

In northern Spain, although students show many similarities with those in other countries, they also exhibit major differences: Spanish students tend not to live on a university campus — unlike the case elsewhere. This means that most interaction takes place in classrooms (Ariño and Llopis, 2011); classes tend to be large, sometimes over a hundred students, making it hard if not impossible to cater to individual needs (Montmarquette et al., 2001); there is little cultural or ethnic diversity and *non-traditional students* [who are more likely to drop-out — as found in other studies, such as those by Stoessel et al. (2015)] are very thin on the ground in Spanish universities. That is because such students tend to drop-out of school and do not make it to Higher Education or opt for Vocational Training instead (University of Sussex, 2015); the link between getting a university degree and a job post-graduation is weaker than in other countries (Prokou, 2008; Schomburg, 2011); few students resort to bank loans to fund their studies and hence the financial disincentives for dropping are not as stark (Hillman, 2014). As Di Pietro (2006) highlighted, dropping out is a phenomenon that is linked to time and setting (even though there may be common features and factors among Higher Education institutions). It therefore behooves universities to constantly update their analyses of the problem.

Accordingly, our study analyses the differences between those students who drop-out and those who stay on. The variables examined for this purpose cover personal, social and academic characteristics that may affect adaptation between student and institution. Regarding the literature review, we assume the following hypothesis:

(a) Students that decided to drop-out have worse integration (social and academic) than those who persist.
(b) Students than quit their university degree migth have worse relationships with teachers and peers than those who do not quit.
(c) Participants behavior (in terms of class attendance, time devoted to study, use of study techniques and performance) would be better in the persistence groups, in comparison to the drop-out group.

## Materials and Methods

### Participants

The research sample was 1,301 students from a university in northern Spain (University of Oviedo). This sample is part of a larger one used in a European project, *The Alfa-Guide Project* (DCI-ALA/2010/94), one of whose lines of action focused on comprehensive diagnosis of the problem of drop-outs in Higher Education. This initiative involved 16 institutions of Higher Education in Europe and America. Parallel research was conducted, the work being coordinated by the Technical University of Madrid. The pooled sample amounted to 9,982 university freshmen in the 2008/9, 2009/10, and 2010/11 academic years. The University of Oviedo took part in the project, contributing 715 participants to the joint sample (of whom 541 were on the drop-out track, while the rest made up the control group). To balance the samples and to perform meaningful analysis of both drop-outs and students who stayed the course, it was decided to broaden the sample to yield a confidence level of 95% and a sampling error not exceeding 3.3%. Specifically, of the 1,301 students in the study, 698 were continuing their studies (36.68% men and 63.32% women) and 603 were dropping out (50.58% men and 49.42% women). The students were drawn from five branches of knowledge (Arts and Humanities, Sciences, Health Sciences, Engineering and Architecture, Social and Legal Sciences).

### Procedure and Instruments

This paper has followed an ex post facto design. The information was gathered in three stages and from two sources. Initially, the university filtered personal information (e.g., age, gender, first year of matriculation, place of residence, etc.) of students who were dropping out. It then chose a control group (students who were staying on) with similar characteristics to the drop-outs. Informed consent was then obtained from each of the students who were to take part in the study. Once consent was given, the third step was for students to answer the *ad hoc*, questionnaire, which was administered remotely (by phone or by e-mail, depending on each student’s preference). The questionnaire applied within the Alfa-Guide project framework consisted of over 100 items, to be completed by the institution and students. It gathered information on the students that was of a demographic, personal, social, institutional, and academic nature. This questionnaire solely covered information on those personal variables bearing on the study objectives. That is to say, it bore on variables linked to university entry, reasons for taking a degree, adaptation to the institution, student behavior in performing academic tasks but discounted performance. Accordingly, the study excluded variables whose natures were demographic, family, institutional and non-academic (for instance, health).

Specifically, the questionnaire used comprised *background* variables, four of them dichotomous (1 = yes, 0 = no) bearing on the reason for the student’s choice of degree (e.g., *The choice of the degree was mainly due to vocational reasons*); a nominal variable on matriculation route (e.g., *I entered university from school etc.*); two nominal variables on the student’s financial means (e.g., *I am financially independent*); and a 5-point Likert scale (1 = very bad/none; 5 = very good/always) covering social-academic data (e.g., *Relationships with peers have been*...); two personal variables on the student’s perception of his academic and social adaptation to the institution; and four academic and personal variables on general performance (e.g., *Rate your level of class attendance*; **Table 1**).

**Table 1:** Summary of variables in this study.

Kind of variable	Item	Possible values
Background	Reason for taking the the degree: *The choice of degree was mainly due to⋅⋅(reasons).*	(i) Vocational (yes/no). (ii) Labor market (yes/no). (iii) Family tradition (yes/no). (iv) Professional orientation (yes/no).
	Path to the degree: *I entered university from:*	University access test or other paths (e.g., Vocational Training, Adult Admission >25 years old).
	Finances: *Financially dependent on:*	Myself or on others (e.g., parents).
Coexistence	Relationship with teachers Relationship with peers Living environment	1 = very bad /none to 5 = very good/always.
Adaptation	Social adaptation Academic adaptation	1 = very bad /none to 5 = very good/always.
Performance	Compliance with program Time devoted to study Use of study techniques Class attendance	1 = very bad /none to 5 = very good/always.

### Data Analysis

Differences between those students who stayed on and those who dropped out were considered in relation to reason for degree choice, the entry path to college and financial dependence. The results were analyzed using the Chi-square test given the dichotomous/nominal nature of the variables used.

The Student’s *t*-test for independent samples was used to see whether there were statistically significant differences between ‘stayers’ and ‘drop-outs,’ depending on the impact of personal, social and academic variables in each case. All the variables met the assumption of normality, following the criterion proposed by Finney and DiStefano (2006) but not all of them met the assumption of homogeneity of variance (Levene test). Accordingly, equal variances in the variables was not assumed. The Effect Size of statistically significant differences was estimated by the *d* Cohen statistic, applying the criteria set out in Cohen’s (1988) seminal work : *d* = 0.20 indicates a small effect size, *d* = 0.50 indicates a medium effect size, *d* = 0.80 indicates a large effect size.

## Results

Regarding students’ first choice of degree, a statistically significant relationship was observed for matriculation being made mainly on vocational grounds (χ^2^ = 45.03; *p* < 0.001), with students who continued their university studies showing a higher percentage for this reason than was the case for drop-outs. As for the other reasons for choice, no statistical differences were observed in any of the cases: *interest in the Labor market* (χ^2^ = 0.75; *p* = 0.386); *family tradition* (χ^2^ = 2.57; *p* = 0.109); and, *professional orientation* (χ^2^ = 1.67; *p* = 0.197).

As for the remaining background variables there are statistically significant differences in both cases — that is to say in both the path to college (χ^2^ = 28.61; *p* < 0.001), being more common for students who stay on to have joined university straight from school, and in relation to financial aspects (χ^2^ = 22.96; *p* < 0.001), being more common for students who are financially independent to show higher drop-out rates.

**Table 2** shows the descriptive results for the group of students that persist and the drop-out group, depending on the personal variables bearing on students’ coexistence in the institution, social and academic adaptation, and general performance. The posible values of these variables rank from 1 to 5. As can be observed, theses means go from 3.5 to 4.41, being “relationship with peers” the one with the highest mean for both groups (persistence and drop-out), also obtaining remarkable puntuations the rest of variables from this group.

**Table 2:** Descriptive statistics of the variables for coexistence, adaptation and performance according to the belonging group (persistent or drop-out).

	Persist (*N* = 698)		Drop-out (*N* = 603)		*DM _S-DO_*	*t*_(gl)_	*p*	*d*
	*M*	*SD*	*M*	*SD*				
**Coexistence**								
Relationship with teachers	4.01	0.58	3.92	0.61	0.087	2.63_(1251)_	0.009	0.15
Relationship with peers	4.41	0.59	4.37	0.59	0.042	1.28_(1299)_	0.200	-
Living environment	4.01	0.59	4.03	0.56	-0.017	-0.52_(1299)_	0.604	-
**Adaptation**								
Social adaptation	4.25	0.57	4.09	0.65	0.166	4.87_(1212)_	0.000	0.26
Academic adaptation	4.03	0.59	3.66	0.77	0.368	9.56_(1121)_	0.000	0.55
**Performance**								
Compliance with program	3.82	0.60	3.74	0.67	0.076	2.14_(1223)_	0.032	0.13
Studying time	3.87	0.73	3.50	0.82	0.373	8.61_(1213)_	0.000	0.48
Use of study techniques	3.81	0.69	3.56	0.76	0.255	6.31_(1224)_	0.000	0.35
Class attendance	4.32	1.03	4.03	1.23	0.280	4.42_(1224)_	0.000	0.26

*M = mean; *SD*, standard deviation. The minimum value of all the variables on the scale is 1 and the maximum value is 5.*

Adaptation (either social or academic) and performance also obtained high puntuations regarding their means. As for the variance within each group, class attendance present the highest standard deviation, showing a relevant variablity in class attendance habtis in both groups.

Results of mean comparison showed that, both, those who presist and those who quit atribute a good value in regard to its social relationships (no diferences statistically significant). No difference was found between the two groups regarding university atmosphere and coexistence or peer relationships. However, this rating is significantly higher in the case of the persistence group. when it comes to the student-teacher relationship, although the effect size is small. In spite of the general perception by students that the relational environment is good, data also reveal how the level of social adaptation to the institution is higher in students who didn’t give up, resulting in statistically significant differences with a small effect size. In other words, whether students continue studying or drop-out, a positive interaction between students and between students and teachers can be observed. That said, the students who drop-out adapt whose than those who stay on. On the other hand, there are statistically significant differences regarding the level of academic adaptation. Again, students who persist show greater adaptation than the drop-outs, with statistically significant differences with a medium size effect.

As for students’ academic performance, there are statistically significant differences between both groups in terms of the time they devoted to studying, the use of study techniques and class attendance. In regard of the size effect, a greater effect was found for study time (medium) and the use of study techniques (low) than for class attendance (low), showing how stayers spend more time working on their own and taking a more strategic approach to academic tasks (that is to say, they adapt better to academic demands). In addition, statistically significant differences between the two groups were observed with regard to program compliance, although the effect size is small.

## Discussion

The beginning of university studies is the turning point in a transition that spans from the start of the course pre-dating college entry to the end of the first year of university (Aguilar, 2007). Both students and institutions need to make social and academic adjustments in the light of the degree program. However, as Tinto (1988) noted, freshers may encounter problems from the outset. If they are not given sufficient support, they may end up adapting poorly to their new university setting. Here, one needs to be aware that many of the variables that affect the drop-out rate lie beyond universities’ control. Two such factors are the student’s socio-economic status and his entry path. That is why it is advisable to focus analysis on those problems where the institution has some leeway (Tinto and Pusser, 2006). That is why, under the European ALFIA-GUIDE project for Drop-Out Management, the University of Oviedo considered studying those variables that might hinder such adaptation between the student and the institution and which the university was in a position to do something about (Marín et al., 2000; Cabrera et al., 2006b; Bethencourt et al., 2008; Elias, 2008; Lehman, 2014). It was also planned for the study to take into account the reasons given by students for their choice of degree, vocation, and the financial and other support provided by the university.

Thus, both so-called *background* variables (such as those bearing on students’ social and academic integration, and general performance) were examined.

As for variables bearing on social integration and adaptation to academic life, three of them (relationship with faculty; level of social adaptation; level of academic adaptation) did not influence either relations with peers or rating of coexistence. This finding may have been colored by the tendency of stayers and drop-outs alike to positively rate both aspects. The results confirm that the relationships forged between teachers and students (when positively rated by the student body) contribute to academic results and the completion of degree studies. These findings are consistent with those obtained by other authors (McPartland and Jordan, 2001; Willcoxson, 2010; Gilardi and Guglielmetti, 2011) and confirm our first and hypothesis. A university is a very different beast from a secondary school not only in terms of academic and administrative size but also with regard to its social scope. Hence the need to ensure student adaptation to this new context. Here, our study has shown that a student is more likely to persevere with his studies if he is well adapted. Similar results were obtained by Tinto (2005), Duncan (2006) and Elias (2008). In this regard, one should note that the World Health Organization (WHO) recommends that education institutions should foster good relations as part of their duty to care for their students’ health and welfare (Prior et al., 2011). This makes it vital to improve university teachers’ initial and continuing training so that faculty members have the knowledge and skills they need to effectively play their tutorial role in the way described by Troyano and García (2011). In this respect, it is also essential that this tutoring role be institutionally acknowledged — something also suggested by Albione et al. (2005).

In connection with the foregoing, it has been found that some paths to university (particularly the one from school) facilitate this adaptation better than others (for instance, professional training, and an entrance exam for those over the age of 25/45). Here, our findings are similar to those obtained by Lassibille and Navarro (2009) and Rodrigo et al. (2012). That is why we recommend special remedial measures be taken for students entering university by paths other than straight from school.

Leaving freshers’ educational backgrounds aside, adaptation to the university setting is a long and often arduous process for many students. This fact makes it advisable to take measures aimed at the student body as a whole. Here, one should note the impact of the passing and application of the University Student Statute (MEC, 2010) in recent years at Spanish universities. The Statute followed on from implementation of the European Higher Education Area (EHEA, 2015), which recognizes students’ rights to tutoring and guidance as part of their education. The Statute has encouraged Spanish universities to set up specific plans of action for tutoring in the various programs offered (in most cases, the faculty draw up these plans). The aim of these plans is to provide career and lifelong guidance, and to monitor student learning (in terms of academic, personal and professional skills). Putting such plans into action is fraught with difficulties given universities’ lack of sufficient resources (Álvarez, 2013; Domínguez et al., 2013). In any event, it is worth faculties drawing up a comprehensive plan of action for student tutorials and monitoring on the lines suggested by Álvarez and González (2009).

As for the variables reflecting the student’s performance (class attendance, time spent studying, use of study skills) and its relation with the studied phenomenon, our findings reveal that only these three variables are linked to dropping out from university: Poor class attendance has been proven to be strongly linked to dropping out from university. This is in line with the results obtained by Iñigo et al. (2011), and Bernardo et al. (2015). Nevertheless, the variable has a low size effect because merely attending classes is no guarantee that the student will benefit from them), as Pintor et al. (2012) highlight. On the other hand, time spent studying/working on one’s own in an assiduous fashion outside exam periods helps shape a student’s study habits and has proven to boost degree completion rates. There is a medium size effect in this case. Similar results were obtained by Elias (2008) and Trevizán et al. (2009) and are supported by the findings of Broadbent and Poon (2015), who (following a systematic review of the relationship between self-regulated learning and academic success) concluded that almost all research studies found a link between time management and academic success.

Likewise, intensive use of study techniques has also been shown to correlate strongly with degree completion. In view of the advanced, specialized content found in modern curriculums, it comes as little surprise that university students can now bring a wider range of learning strategies and study techniques to bear in their academic work. These findings are consistent with those of Bethencourt et al. (2008), who affirm that this of variables play a remarkable role in dropping out of university.

Thus, students who persist spend more time working on their own and do a better use of study techniques (in line with our hypothesis). Such students are more authonomous in the teaching-learning process, which confirms that training measures focusing on these skills will yield better academic results (Tan et al., 2008; Balkıs, 2011). For example, Azevedo et al. (2010) proposed the use of MetaTutor software, which purpose is to provide diagnosis and training for self-learning in virtual settings. The software also allows one to broaden scientific knowledge of these highly popular environments and provides a useful tool for greatly boosting students’ academic performance.

One of the questions that now arises is how to put theory into practice, i.e., get the student’s retention in university classrooms. Given the results of our research, it seems that the institution as a whole, and its role in the EHEA’s work, are both on the right track. However, one must also give students tools to help them adapt academically and its diverse demands. The introduction of hosting programs (to facilitate students’ initial adaptation; commitment to their degrees; practice in training strategies; time management) all help boost academic performance and completion of studies. However, at this point one should recall the recommendation made by Tinto and Pusser (2006) on the need to systematically tackle the issue of university drop-outs. All remedial measures need to form part of an Institutional Action Plan that involves the various groups and ensures proper resource management. The aim should be to exploit synergies to render plan implementation more effective. Nevertheless, the greatest limitations here stem from the savage cuts that have been made in Spanish universities since the beginning of the present economic crisis makes it hard to implement such plans and research into the problem of university drop-out.

Future research might employ a representative sample of students from other universities operating in similar cultural settings and analyze whether the results here are consistent with those found in other branches of knowledge.

## Limitations and Future Reseach

This paper show results obtained in an ex post facto research. Althougth this research method have important limitations, such as the inability to manipulate the independent variables, our research team considered that was the most suitable design; it is not practical to apply experimental design to study university drop-out, as this kind of design would oblige us to wait at least 1 year between the aplication of the pre-test and the post-test, in order to wait for the phenomenon to occur. However, the cost and time savings, result of this kind of research, are remarkables advantages that we took into account.

It is also neccessary highlight that in this research we have not explored in depth the psychological characteristics of our participants and are often related to drop-out (eg., self-efficacy, resilience, mental health) due to budget and time limitations. Therefore, it would be advisable to develop further research to analyze the influence of these variables in the phenomenon.

## Author Contributions

AB directed the research developed in the frame of the Alfaguia Project (funded by the European Union) and was one of the authors of this paper. The other authors are: ME, who was the student that assisted the director along every research phase and still contributing in this research topic. AC, who is a PhD student that is developing his thesis about university drop-out, using the data collected in Alfaguia Project and, therefor, is a contributor of this paper. EF, ET, and PS are team members that joint this research topic once that Alfaguia Project was finished, but contribute to study university abandonment helping AB reviewing the literature, carrying out analysis and writing papers.

## Conflict of Interest Statement

The authors declare that the research was conducted in the absence of any commercial or financial relationships that could be construed as a potential conflict of interest.
