# Corrigendum: Comparison of Personal, Social and Academic Variables Related to University Drop-out and Persistence

**DOI:** 10.3389/fpsyg.2017.01355

**Published:** 2017-08-02

**Authors:** Ana Bernardo, María Esteban, Estrella Fernández, Antonio Cervero, Ellián Tuero, Paula Solano

**Affiliations:** ^1^Psychology Department, University of Oviedo Oviedo, Spain; ^2^Department of Education, University of Oviedo Oviedo, Spain

**Keywords:** higher education, university drop-out, academic performance, academic adaptation, social adaptation

There is an omission on the funding section, as authors specified the project funds, but did not mention additional funds. Therefore, where in the original article says;

“Alfaguia Project was developed thanks to the European Union funding (DCI-ALA/2010/94).”

Should say:

“Alfaguia Project was developed thanks to the European Union funding (DCI-ALA/2010/94). In addition, our research activity is also granted by European Regional Development Funds (European Union and Principality of Asturias) through the Science, Technology and Innovation Plan (GROUPIN14-100 and GROUPIN14-053).”

The authors apologize for any caused inconvenience. This omission does not affect the scientific conclusions of the article.

## Conflict of interest statement

The authors declare that the research was conducted in the absence of any commercial or financial relationships that could be construed as a potential conflict of interest. The handling Editor declared a shared affiliation, though no other collaboration, with the authors AB, ME, EF, AC, ET, and PS and states that the process nevertheless met the standards of a fair and objective review.

